# Primary Multifocal Hepatic Gastrointestinal Stromal Tumor Mimicking Metastatic Disease: A Diagnostic Challenge With PET-CT and Histopathological Correlation

**DOI:** 10.7759/cureus.107964

**Published:** 2026-04-29

**Authors:** Rohan G Padia, Rajoo Ramachandran, Venkata Sai, Perarivalan Ilango

**Affiliations:** 1 Radiology and Imaging Sciences, Sri Ramachandra Institute of Higher Education and Research, Chennai, IND; 2 Radiology, Sri Ramachandra Institute of Higher Education and Research, Chennai, IND; 3 Radiodiagnosis, Shri Sathya Sai Medical College, Chennai, IND

**Keywords:** cd117, extra-gastrointestinal stromal tumor, gastrointestinal stromal tumor, hepatic lesion, imatinib response, multifocal, primary hepatic gastrointestinal stromal tumor, primary hepatic gist

## Abstract

Primary hepatic gastrointestinal stromal tumor (PHGIST) is an exceptionally rare extra-gastrointestinal stromal tumor arising within the liver. It poses a significant diagnostic challenge because hepatic involvement in gastrointestinal stromal tumor (GIST) is more commonly metastatic. Multifocal primary hepatic presentation is even less frequently described. We report a case of a 62-year-old male presenting with constitutional symptoms, wherein contrast-enhanced computed tomography (CECT), magnetic resonance imaging (MRI), and PET-CT revealed multiple lobulated solid lesions involving the right lobe of the liver, associated with mild circumferential thickening of the gastroesophageal junction (GEJ). The lesions demonstrated internal septations, mural nodules, diffusion restriction, subtle wall enhancement, and fluorodeoxyglucose (FDG) avidity, initially raising concern for metastatic disease in the liver. However, histopathological examination of ultrasound-guided liver biopsy revealed a spindle cell neoplasm arranged in sheets and fascicles, with tumor cells showing strong diffuse CD117 positivity on immunohistochemistry. A separate upper gastrointestinal endoscopy-guided biopsy from the GIST demonstrated only mild hyperplasia without evidence of dysplasia or malignancy. The combined radiologic and pathologic findings favored a diagnosis of primary multifocal hepatic GIST. On follow-up PET-CT scan after imatinib therapy, the patient showed a complete/near complete metabolic response. This report expands the imaging spectrum of PHGIST and emphasizes the importance of radiologic-pathologic correlation in distinguishing this rare lesion from metastatic liver disease.

## Introduction

Gastrointestinal stromal tumors (GISTs) are the most common mesenchymal neoplasms of the gastrointestinal tract, arising from the interstitial cells of Cajal or related precursor stem cells, which serve as pacemaker cells regulating gut motility [1,2]. These tumors most frequently occur in the stomach (50-60%), followed by the small intestine (30-35%), while involvement of the colon and rectum (approximately 5%) is relatively uncommon [1,2]. Histologically, GISTs are typically classified into spindle cell, epithelioid, or mixed subtypes, and on immunohistochemistry, they characteristically express KIT (CD117), which remains the cornerstone of diagnosis [2].

A small subset of these tumors arises outside the gastrointestinal tract and is designated as extra-gastrointestinal stromal tumors (EGISTs), which comprise about <5% of all GISTs [3]. Extra-gastrointestinal sites include the omentum, mesentery, retroperitoneum, pancreas, gallbladder, and rarely the liver [3,4]. Among these, primary hepatic gastrointestinal stromal tumor (PHGIST) represents one of the rarest forms, comprising only about <1% of all GIST cases, with only isolated case reports and literature reviews available to date [3-6].

The diagnosis of PHGIST is particularly challenging because multifocal hepatic involvement is more indicative of a metastatic lesion than primary origin. Therefore, primary hepatic GIST is a diagnosis of exclusion, and other differentials can be ruled out through cross-sectional imaging, histopathologic correlation, and immunohistochemistry, along with molecular diagnostic studies [3,5].

Radiologically, PHGISTs have been typically described as a solitary large solid mass, usually involving a single hepatic lobe [3-6]. The present report is noteworthy because it expands the known imaging spectrum of PHGIST, highlights the importance of radiologic-pathologic correlation, and emphasizes that multifocal solid liver lesions may not always be metastatic [5,6].

## Case presentation

A 62-year-old male with a history of chronic alcohol intake presented on 09/08/2025 with generalized weakness, decreased appetite, and unquantifiable weight loss for one month. There was no history of vomiting, nausea, or altered bowel habits. The patient’s biochemical profile revealed markedly elevated alkaline phosphatase levels and mildly elevated serum glutamic oxaloacetic transaminase (SGOT) and serum glutamic pyruvic transaminase (SGPT) levels (Table 1).

**Table 1:** Laboratory evaluation.

Parameter	Value	Reference
Hemoglobin	14 g/dL	12-16 g/dL
Total leukocyte count	10,060/mm³	4-11 x 10³/mm³
Alkaline phosphatase	375 IU/L	40-129 IU/L
Serum glutamic oxaloacetic transaminase (SGOT)	54 U/L	<40 U/L
Serum glutamic pyruvic transaminase (SGPT)	64 U/L	<41 U/L
Gamma glutamyltransferase	272 U/L	<60 U/L
Total protein	6.9 gm/dL	6.6-8.7 gm/dL
Serum albumin	3.5 gm/dL	3.97-4.94 gm/dL
Serum globulin	3.4 gm/dL	2-3.5 gm/dL
Albumin-to-globulin ratio	1	1.1-2
Total bilirubin	0.46 mg/dL	<1.2 mg/dL
Direct bilirubin	0.36 mg/dL	<0.2 mg/dL
Indirect bilirubin	0.1 mg/dL	0.1-0.8 mg/dL
Carcinoembryonic antigen (CEA)	0.73 ng/mL	<3 ng/mL
CA 19.9	1.38 U/mL	<34 U/mL
Alpha fetoprotein (AFP)	1.33 ng/mL	<7 ng/mL

USG findings

**Figure 1:**
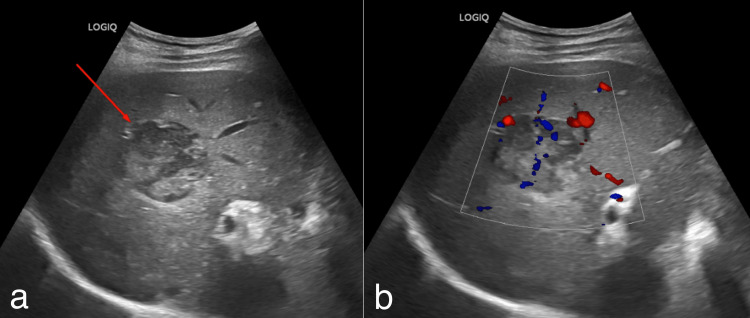
Ultrasound of the abdomen. A few relatively well-defined heteroechoic lesions are noted in the right lobe of the liver, indicated by the red arrow (a), showing mild internal and peripheral vascularity on color Doppler (b).

Abdominal ultrasonography (USG) done on 09/08/2025 revealed a few ill-defined heteroechoic solid lesions in the right lobe of the liver, the largest measuring 5 × 5 cm, located in segment VIII (Figure 1).

Contrast-enhanced CT findings

**Figure 2:**
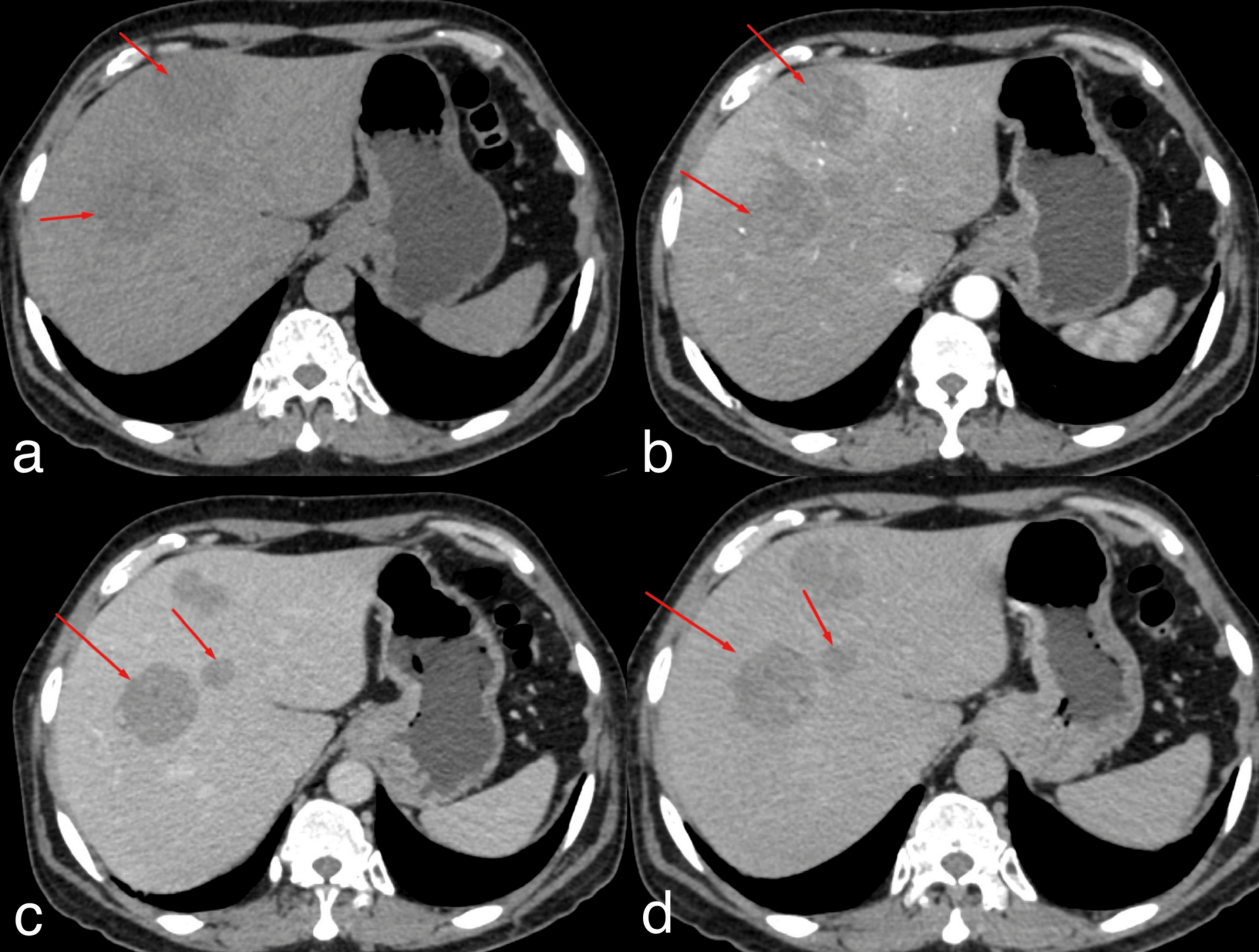
CECT of the abdomen. Contrast-enhanced computed tomography (CECT) showing multiple (four to five) well-defined lobulated heterogeneously hypodense lesions in the plain (a) and hypoenhancing lesions in the arterial phase (b) and venous phase (c) without appreciable washout in the delayed phase (d) in the right hepatic lobe, indicated by red arrows.

Contrast-enhanced computed tomography (CECT) of the abdomen was performed on 12/08/2025, which revealed multiple (four to five) well-defined lobulated heterogeneously hypodense and hypoenhancing lesions without appreciable washout involving segments IVb, VII, and VIII, predominantly in the right hepatic lobe, with a few lesions in subcapsular location. The largest lesion in segment VIII measured 5.5 × 5.1 × 4.8 cm (Figure 2).

Contrast-enhanced MRI findings

**Figure 3:**
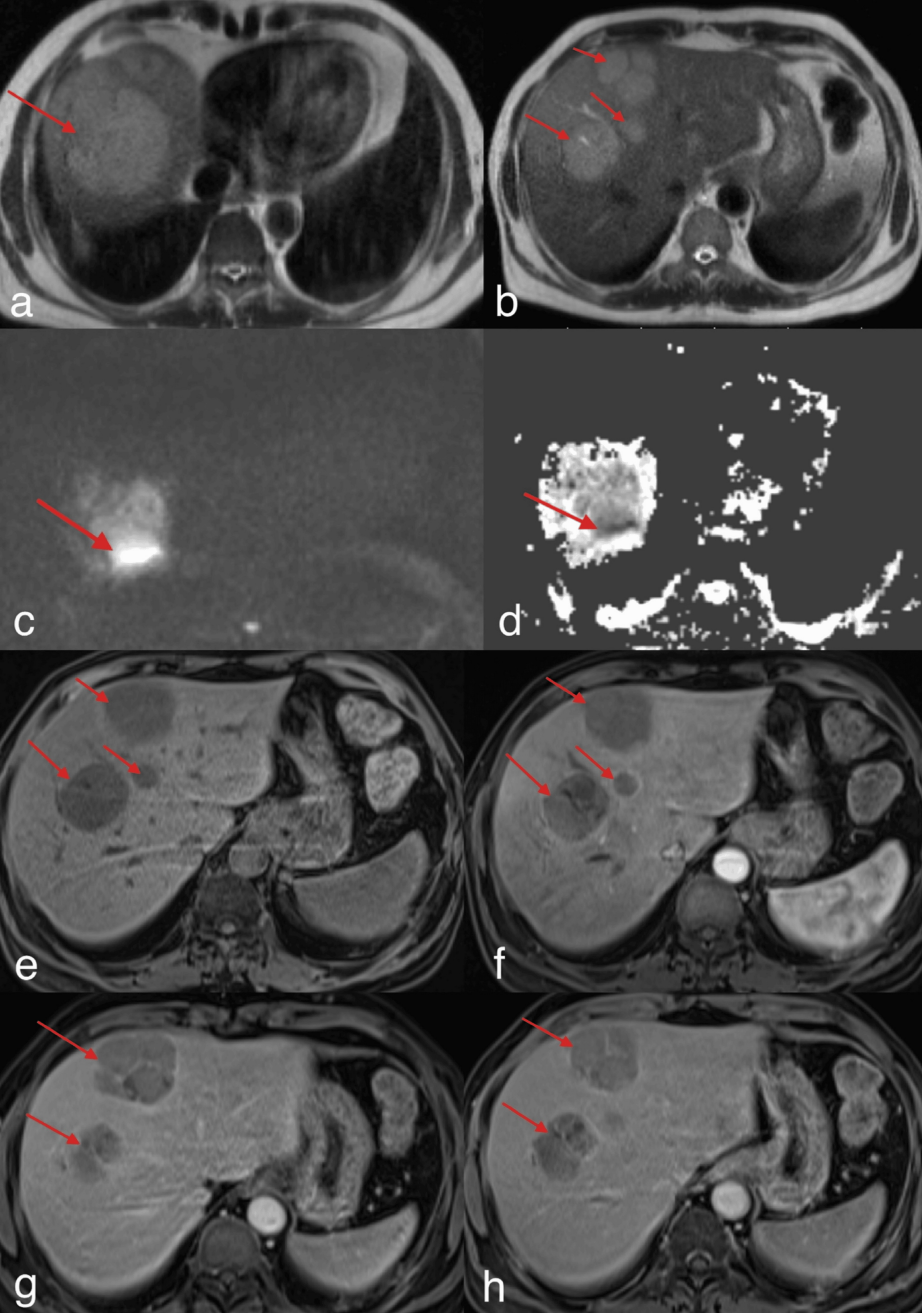
CE-MRI of the abdomen. Contrast-enhanced MRI (CE-MRI) showing lobulated T2 hyperintense lesions (a, b) showing peripheral diffusion restriction on diffusion-weighted imaging (DWI)/apparent diffusion coefficient (ADC) map (c, d) and T1 hypointense signal (e) with subtle peripheral and septal enhancement on post-contrast images in the arterial phase (f), venous phase (g), and delayed phase (h), indicated by red arrows.

Subsequent MRI of the upper abdomen done on 19/08/2025 showed approximately five lobulated hepatic lesions seen in segments VIII, IVa, and VII, showing T2 hyperintense and T1 hypointense signal with peripheral and septal post-contrast enhancement with a few mural nodules. Peripheral and septal diffusion restriction was seen. No vascular invasion, ascites, or significant abdominal lymphadenopathy was present (Figure 3).

On CT and MRI, the imaging differentials considered included: (1) primary esophageal malignancy with hepatic metastases; (2) hepatic epithelioid hemangioendothelioma.

Histopathological findings

**Figure 4:**
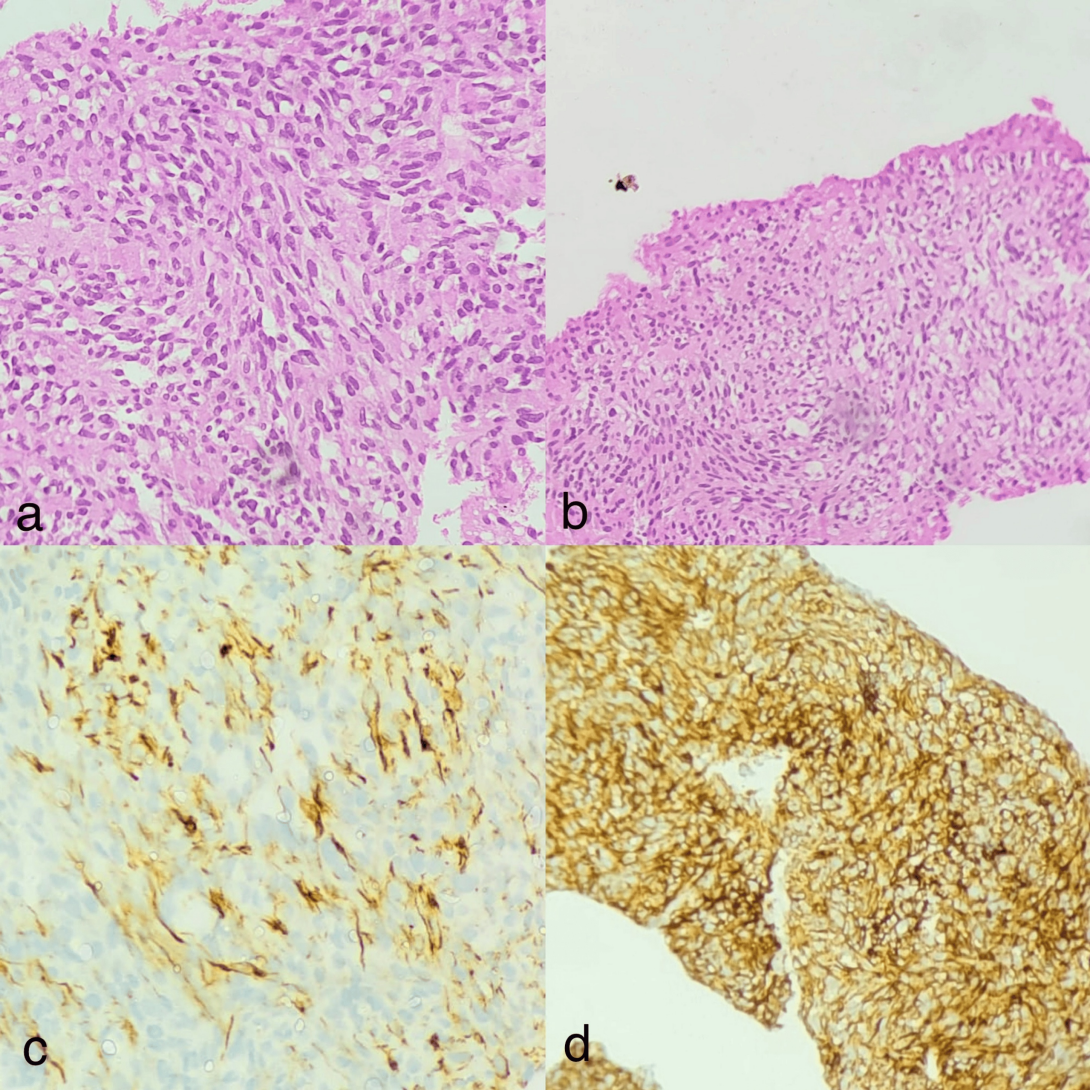
Histopathological and immunohistochemical analysis of liver biopsy. Histopathological examination of liver biopsy revealed spindle-shaped tumor cells arranged in sheets and fascicles with tumor cells showing elongated hyperchromatic nuclei with moderate eosinophilic cytoplasm and focal cytoplasmic vacuolation (Image a - HE stained, 40x magnification; Image b - HE stained, 10x magnification) and immunohistochemistry demonstrating focal desmin positivity (Image c - Desmin marker, 40x magnification) and strong diffuse CD117 positivity (Image d - CD117 marker, 10x magnification). HE: hematoxylin and eosin.

Histopathological examination of USG-guided liver biopsy performed on 20/08/2025 revealed linear cores of liver tissue infiltrated by spindle-shaped tumor cells arranged in sheets and fascicles, interspersed by blood vessels. The tumor cells showed elongated hyperchromatic nuclei with moderate eosinophilic cytoplasm and focal cytoplasmic vacuolation. Immunohistochemistry demonstrated strong diffuse CD117 positivity, focal desmin positivity, negative pancytokeratin (panCK), negative ERG, and negative p40. These findings were in favor of GIST (Figure 4).

PET-CT findings

**Figure 5:**
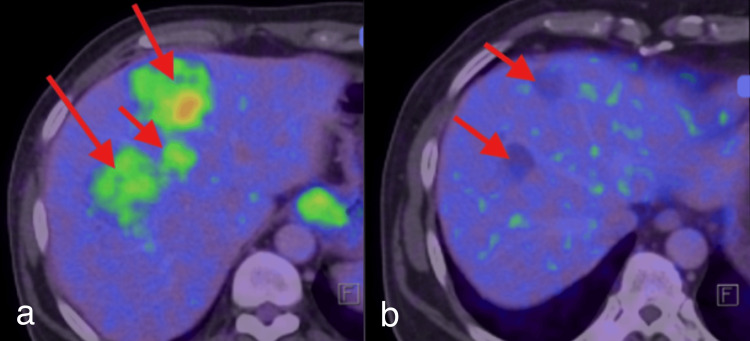
PET-CT of the whole body. PET-CT showed multiple fluorodeoxyglucose-avid lesions (red arrows) (a) in the right lobe of the liver. After imatinib therapy for five months, the lesions showed complete metabolic resolution with significant size resolution in the follow-up PET-CT (red arrows) (b).

Initial whole-body 18F-fluorodeoxyglucose (18F-FDG) PET-CT evaluation done on 04/09/2025 showed multiple FDG-avid (maximum standardized uptake value (SUVmax) = ~12.1) solid lesions in the right lobe of the liver, the largest measuring 81 x 74 mm in segment VIII, suggestive of multifocal metabolically active hepatic neoplastic lesions. Follow-up whole-body 18F-FDG PET-CT evaluation done on 18/02/2026 after imatinib therapy for five months showed complete metabolic resolution with significant size resolution of the lesions, with the largest lesion in segment VIII now measuring 45 x 35 mm after imatinib therapy (Figure 5).

**Figure 6:**
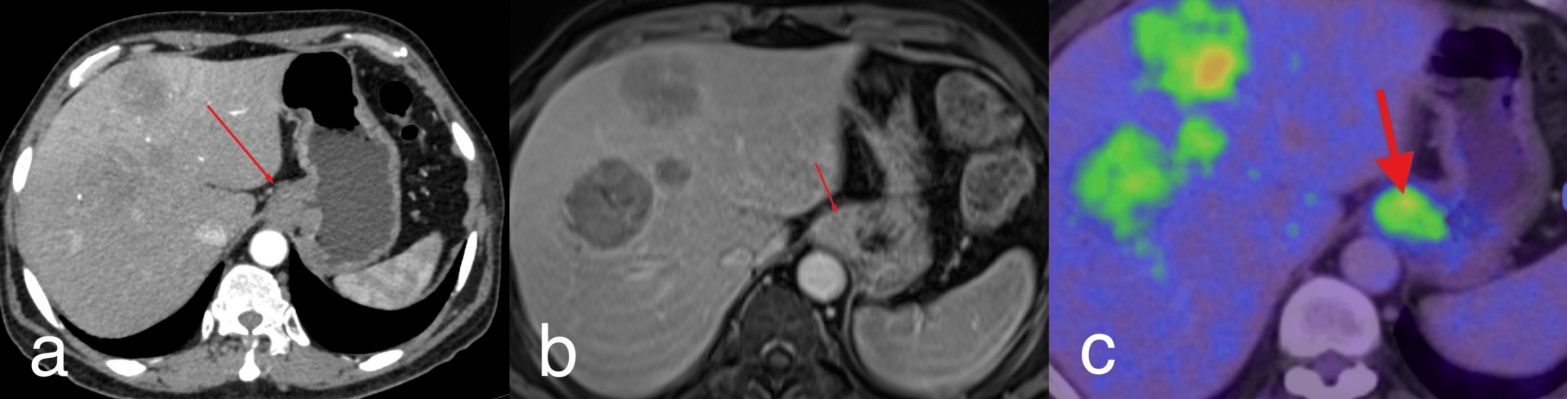
Circumferential enhancing wall thickening. Circumferential enhancing wall thickening at the gastroesophageal junction is seen on contrast-enhanced CT (a) and contrast-enhanced MRI (b), along with fluorodeoxyglucose avidity (red arrow) in PET-CT (c).

Additionally, circumferential enhancing irregular wall thickening of the gastroesophageal junction (GEJ) showed mild diffusion restriction on MRI and FDG avidity in PET-CT, with a maximal single wall thickness of 13 mm, with mild luminal narrowing but preserved oral contrast passage into the stomach. No upstream esophageal dilatation, perigastric fat stranding, or locoregional lymphadenopathy was identified, and a possibility of neoplastic etiology was raised (Figure 6).

**Figure 7:**
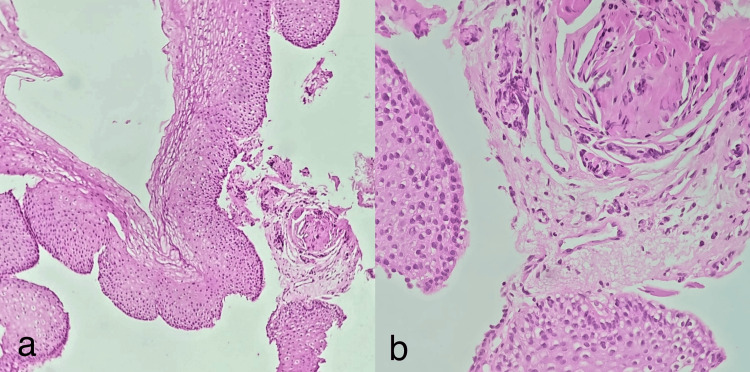
Histopathological examination of GEJ thickening. Histopathological examination of gastroesophageal junction (GEJ) thickening biopsy demonstrating only mild hyperplasia without evidence of dysplasia, granuloma, or malignancy (Image a - HE stained, 10x magnification) (Image b - HE stained, 40x magnification). HE: hematoxylin and eosin.

Importantly, histopathological examination of endoscopy-guided biopsy from the GEJ thickening done on 14/08/2025 demonstrated only mild hyperplasia without evidence of dysplasia, granuloma, or malignancy (Figure 7).

The patient did not undergo any colonoscopy or endoscopic ultrasound examination.

## Discussion

PHGIST is an exceptionally rare form of EGIST, accounting for a very small fraction of all GISTs. In the study done by Qian et al., the majority of reported hepatic GISTs were solitary lesions, with multifocal liver disease being distinctly uncommon [6]. The present case is particularly significant because it demonstrates an unusual multifocal primary hepatic presentation.

The patient presented with generalized weakness, decreased appetite, and unquantifiable weight loss for one month with a history of chronic alcohol intake. The patient’s biochemical profile revealed altered liver function tests (LFT) showing markedly elevated alkaline phosphatase levels and mildly elevated SGOT and SGPT levels, indicating hepatic involvement.

From an imaging standpoint, the lesions in our case significantly broaden the recognized radiologic spectrum of PHGIST. On CT, the masses were well-defined, with lobulated hypoenhancing lesions without appreciable washout, while MRI demonstrated T1 hypointensity, T2 hyperintensity, internal septations, mural nodularity, peripheral and septal diffusion restriction, and subtle wall and septal enhancement.

Previously published hepatic GISTs commonly demonstrate T1 hypointensity, T2 hyperintensity, heterogeneous enhancement, and areas of cystic degeneration or hemorrhage, findings largely attributable to intratumoral necrosis and secondary degenerative changes [4,5]. However, the multiloculated solid morphology with septations and mural nodules in our patient is particularly deceptive and closely simulates metastases, hepatic epithelioid hemangioendothelioma (HEHE), or necrotic cholangiocarcinoma.

Histopathologically, sections studied from the hepatic lesion revealed spindle cell morphology arranged in sheets and fascicles with intervening vascular channels, a classic microscopic pattern described in GIST [1,7]. Strong diffuse CD117 positivity on immunohistochemistry provided crucial diagnostic confirmation, as KIT expression is seen in approximately 95% of classic GISTs [7,8]. The diagnosis is supported by CD117 positivity in an appropriate spindle cell neoplasm, together with negative epithelial markers (panCK, p40) and negative vascular marker ERG, which is highly characteristic of GIST [7,8]. The focal desmin positivity seen in this case does not exclude GIST and has been described occasionally. Evaluation of DOG1, CD34, SMA, S100, and Ki-67 immunohistochemistry markers was not performed in this patient, and this limitation should be acknowledged in the given study.

A major diagnostic challenge in this case was the coexisting GEJ thickening, which initially raised the possibility of a primary upper gastrointestinal malignancy with hepatic metastases. However, several imaging features argued against an aggressive GEJ primary lesion: no upstream esophageal dilatation, preserved perigastric fat planes, absence of locoregional lymphadenopathy, intact adjacent serosa, and no perigastric inflammatory stranding. These findings were confirmed by histopathological examination of an endoscopic GEJ biopsy, which revealed only mild hyperplasia without evidence of dysplasia or malignancy, excluding a primary lesion at that site. This remains one of the strongest academic points of this report because it supports a more confident diagnosis of primary hepatic origin.

The PET-CT findings further strengthened the imaging correlation by demonstrating multiple FDG-avid hypoenhancing solid lesions involving the liver, supporting the presence of metabolically active viable tumor tissue. No metabolically active lesion other than the hepatic lesions and GEJ thickening was identified on whole body PET-CT, ruling out the possibility of hepatic metastasis.

On follow-up PET-CT scan after imatinib therapy, the patient showed a complete metabolic response.

The case, therefore, highlights that multifocal solid liver lesions may not always be metastatic. The radiological-pathological correlation favored the diagnosis of primary hepatic GIST after no definite gastrointestinal primary lesion was identified.

## Conclusions

The diagnosis of primary hepatic GIST was favored after radiologic-pathologic correlation and absence of an identifiable gastrointestinal primary lesion on available evaluation. This case highlights the importance of considering rare mesenchymal neoplasms in the differential diagnosis of multifocal solid hepatic lesions, particularly when no definite gastrointestinal primary lesion is identified. Definitive diagnosis relies on meticulous radiologic-pathologic correlation and immunohistochemistry, which are essential for the timely initiation of targeted therapy and a favorable clinical response.

## References

[1] Miettinen M, Lasota J (2006). Gastrointestinal stromal tumors: pathology and prognosis at different sites. Semin Diagn Pathol.

[2] Joensuu H, Hohenberger P, Corless CL (2013). Gastrointestinal stromal tumour. Lancet.

[3] Luo XL, Liu D, Yang JJ, Zheng MW, Zhang J, Zhou XD (2009). Primary gastrointestinal stromal tumor of the liver: a case report. World J Gastroenterol.

[4] Nagai T, Ueda K, Hakoda H (2016). Primary gastrointestinal stromal tumor of the liver: a case report and review of the literature. Surg Case Rep.

[5] Cheng X, Chen D, Chen W, Sheng Q (2016). Primary gastrointestinal stromal tumor of the liver: a case report and review of the literature. Oncol Lett.

[6] Qian XH, Yan YC, Gao BQ, Wang WL (2020). Prevalence, diagnosis, and treatment of primary hepatic gastrointestinal stromal tumors. World J Gastroenterol.

[7] Hirota S, Isozaki K, Moriyama Y (1998). Gain-of-function mutations of c-kit in human gastrointestinal stromal tumors. Science.

[8] Rubin BP, Heinrich MC, Corless CL (2007). Gastrointestinal stromal tumour. Lancet.

